# Supplementary material for: Hydrolysis of Extracellular ATP by Vascular Smooth Muscle Cells Transdifferentiated into Chondrocytes Generates Pi but Not PPi
Source: Int J Mol Sci. 2021 Mar 14;22(6):2948. doi: 10.3390/ijms22062948 (PMC8000465; doi:10.3390/ijms22062948)
Supplement: Supplementary file 1 [file ijms-22-02948-s001.pdf]

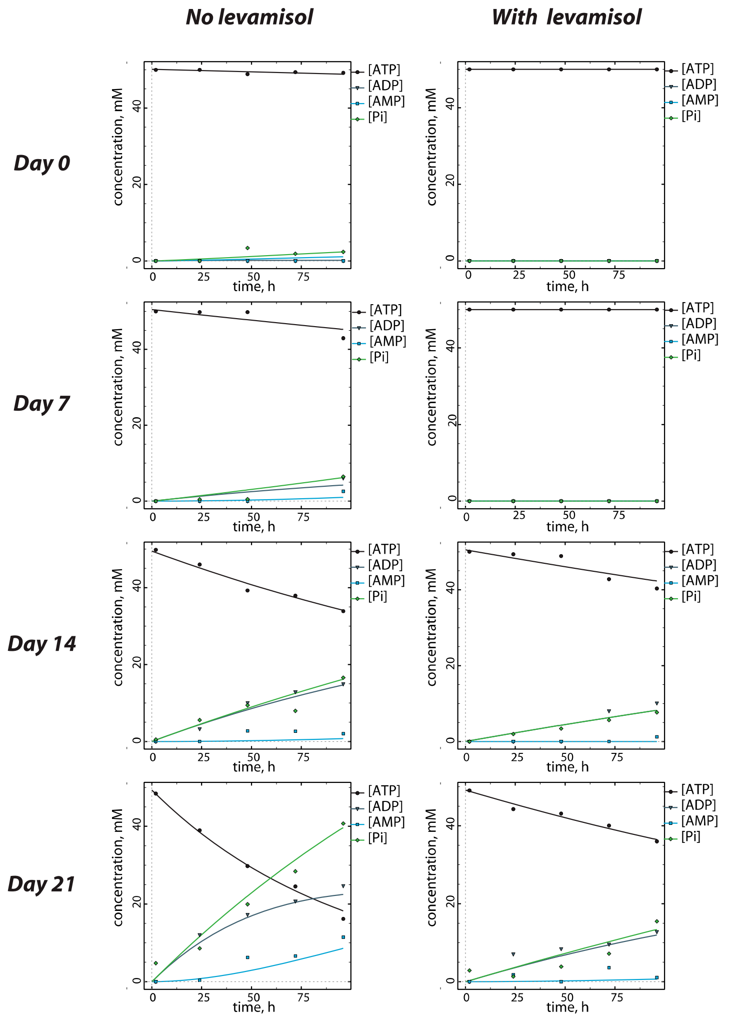

Figure S1. The amount of ATP, ADP, AMP, and P_i in the extracellular medium extracted from MOVAS cells as determined using ^{31}P-NMR. MOVAS cells were incubated for 0, 7, 14, or 21 days in osteogenic medium (denoted Day 0, Day 7, Day 14, and Day 21, respectively). Then, they were washed, fixed, and incubated with 50 mm ATP without (left panel) or with 5 mm levamisole (right panel) for 2, 24, 48, 72, and 96 h. Symbols are the amounts of nucleotides and phosphate determined by ^{31}P-NMR. Full lines correspond to the best fit, which allowed the kinetic constants to be determined.

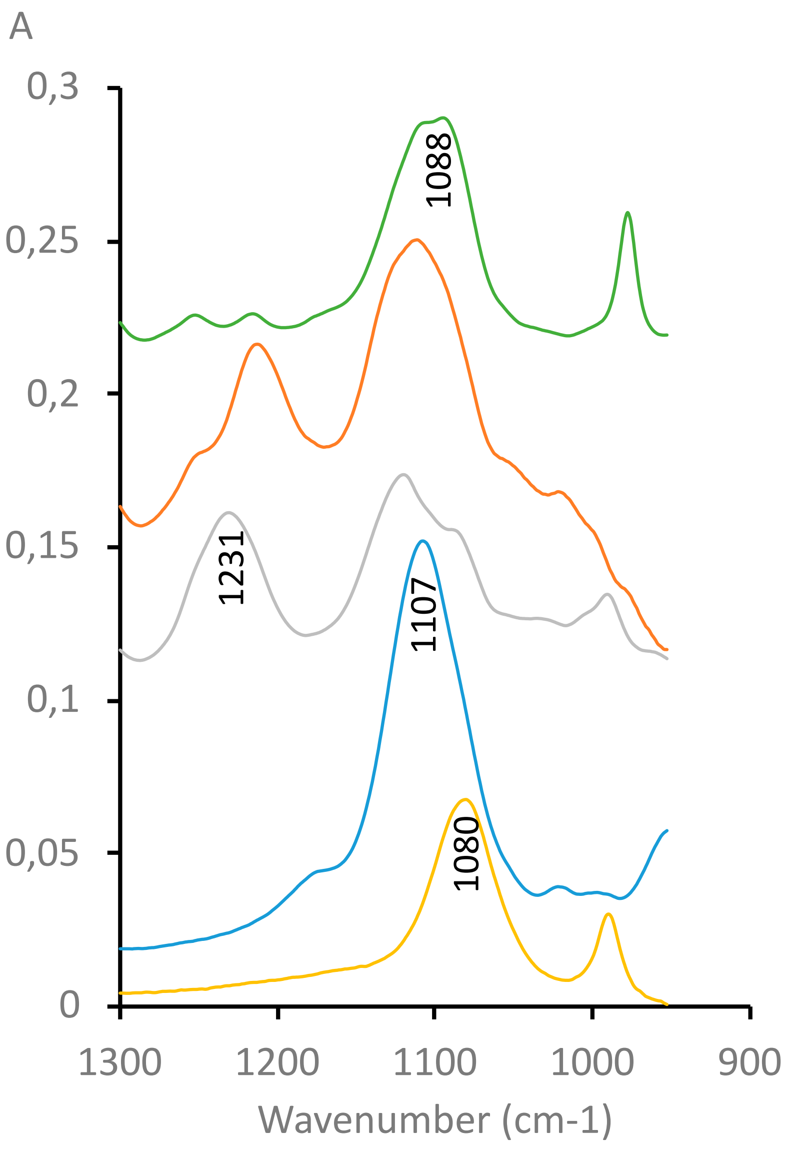

Figure S2. IR spectra of 50 mm AMP (green), ADP (orange), ATP (grey), PP_i (blue), and P_i (yellow) in aqueous Tris buffer (pH 7.8, 100 mm Tris, 5 mm $MgCl_2$, 5 µm $ZnCl_2$) in the region of phosphate vibrational modes. The spectra are upscaled from each other for better visibility (Pathlength of the cell was 12 µm).

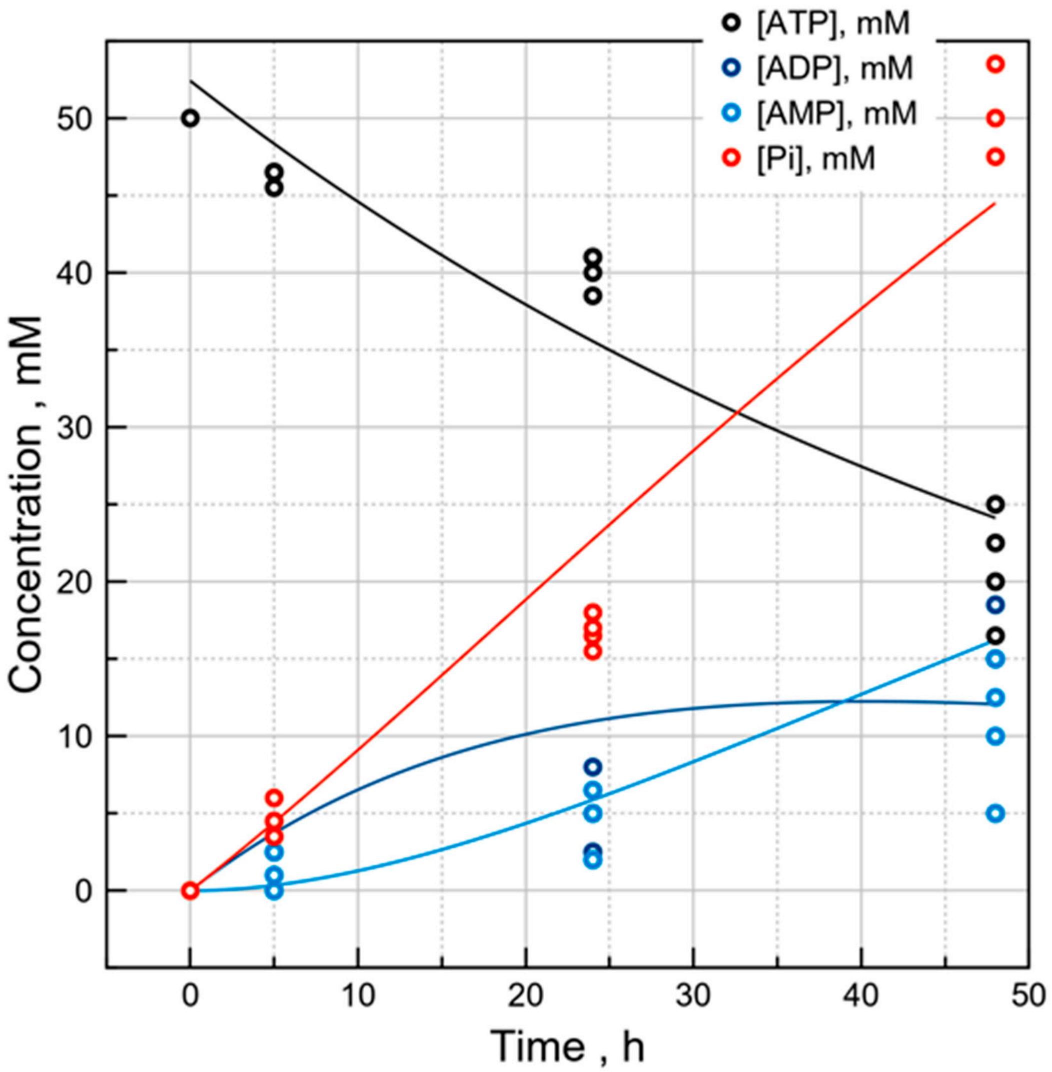

Figure 3. The amount of ATP, ADP, AMP, and P_i in the extracellular medium extracted from MOVAS cells as determined using IR. MOVAS cells were incubated for 14 days in osteogenic medium. Then, they were washed, fixed, and incubated with 50 mm ATP for 5, 24, 48 h. Full lines correspond to the best fit, which allowed the kinetic constants to be determined.
